# What’s new in surgery? Essentials 2024 – young patients and fast decisions

**DOI:** 10.1515/iss-2024-0038

**Published:** 2024-11-15

**Authors:** Juliane Kröplin

**Affiliations:** Department of Oral and Maxillofacial Surgery, 39071 University Hospital of Rostock 18057, Rostock, Germany

Dear colleagues,

I am pleased to present the second issue of “What’s new in Surgery”. In 2024, we have once again put together a surgical interdisciplinary portfolio of the latest surgical evidence for you. This time, the focus is on our youngest patients and surgical treatment approaches whose success depends crucially on making the right decision at an early stage.

Pediatric surgery colleagues Sabine Drossard and Louisa Schuffert will kick off the session with their review of the current evidence on early enteral nutrition (EEN) following intestinal anastomosis in pediatric patients. According to this, a better outcome of young patients and a shorter hospital stay after abdominal surgery can be achieved if individualized feeding initiation and standardized postoperative feeding protocols are used.

The article by Friedrich et al. deals with the surgical treatment of jaw malpositions and presents knowledge and digital approaches in orthognathic surgery. Anomalies of jaw position and shape affect approximately 10 % of the population and are often congenital. Innovative technologies such as VSP, AI and PSIs are increasingly becoming the focus of orthognathic surgical interventions in order to increase the accuracy of surgical results. However, the digital transformation of surgical planning also brings new challenges.

Schlottmann and Lorbeer provide an insight into the complex treatment of patients with burn injuries and highlight the particular importance of specialized burn centers with established treatment standards. In particular, however, technical achievements and new materials for skin replacement still need to be critically evaluated with regard to their clinical applicability.

In their review “Periprosthetic joint infections – a scoping review” Youssef et al. show that fast diagnosis is also of particular importance for adequate treatment in patients with periprosthetic joint infection. However, the correct therapy is still the subject of controversial discussions and requires further evidence.

I hope you enjoy reading the contributions in this issue.

